# IgMAT: immunoglobulin sequence multi-species annotation tool for any species including those with incomplete antibody annotation or unusual characteristics

**DOI:** 10.1186/s12859-023-05624-2

**Published:** 2023-12-21

**Authors:** Daniel Dorey-Robinson, Giuseppe Maccari, John A. Hammond

**Affiliations:** 1 The Pirbright Institute, Pirbright, UK; 2 Anthony Nolan Research Institute, London, UK

**Keywords:** Immunoglobulin, Annotation, IMGT

## Abstract

**Background:**

The advent and continual improvement of high-throughput sequencing technologies has made immunoglobulin repertoire sequencing accessible and informative regardless of study species. However, to fully map dynamic changes in polyclonal responses precise framework and complementarity determining region annotation of rearranging genes is pivotal. Most sequence annotation tools are designed primarily for use with human and mouse antibody sequences which use databases with fixed species lists, applying very specific assumptions which select against unique structural characteristics. For this reason, data agnostic tools able to learn from presented data can be very useful with new species or with novel datasets.

**Results:**

We have developed IgMAT, which utilises a reduced amino acid alphabet, that incorporates multiple HMM alignments into a single consensus to automatically annotate immunoglobulin sequences from most organisms. Additionally, the software allows the incorporation of user defined databases to better represent the species and/or antibody class of interest. To demonstrate the accuracy and utility of IgMAT, we present analysis of sequences extracted from structural data and immunoglobulin sequence datasets from several different species.

**Conclusions:**

IgMAT is fully open-sourced and freely available on GitHub (https://github.com/TPI-Immunogenetics/igmat) for download under GPLv3 license. It can be used as a CLI application or as a python module to be integrated in custom scripts.

**Supplementary Information:**

The online version contains supplementary material available at 10.1186/s12859-023-05624-2.

## Background

Whole antibody repertoire sequencing can result in millions of sequences which can require various layers of filtering for specificity and quality. Perhaps the most important step is accurate annotation of the framework (FR) and complementary determining (CDR) regions (FR1-4 and CDR1-3) that underpins the accuracy of most downstream analyses. Whilst there are numerous web servers available with such functionality, the ability to run a tool locally as part of an in-house workflow is required for many projects. One such too is ANARCI [4], which applies a range of numbering schemes to annotate input sequences by applying Hidden Markov Models (HMMs) trained with curated data from the IMGT database [7]. However, this approach does not provide adequate flexibility to annotate antibody sequences from species with unusual structural properties. This is problematic where species have incomplete IMGT records or use genetic mechanisms that may inhibit alignments to standard gene sequences such as gene conversion in chickens [2]. Further, tools which were designed using assumptions based on model species such as human and mouse inefficiently capture or exclude unusual antibodies, for example imposing CDRH3 maximum length fails to identify ultralong antibodies in cattle [3].

Here we present IgMAT (Immunoglobulin Multispecies Annotation Tool), a tool for the automatic discrimination and annotation of the FR and CDR regions from antibody amino acid sequences, specifically designed to be integrated into custom analysis pipelines. IgMAT is based on the ANARCI tool, with extended capability to annotate antibody sequences from multiple species. The tool is highly customizable, allowing the addition of custom antibody sequence datasets and generating a range of output formats including a bed file of FR and CDR coordinates, enabling downstream analyses as required.

## Implementation

IgMAT provides convenient tools for the analysis and annotation of antibody sequences, allowing the analysis of multiple sequences at the same time. Like many other antibody numbering tools (ANARCI, PyIgClassify, ProABC [1, 4, 13]), the algorithm applies a set of precomputed HMMs to align the input sequences according to the IMGT numbering schemes (MP [8] and successively perform annotation. By default, IgMAT uses a dataset of curated germline antibody sequences of different domains for a set of organisms from the IMGT/Gene Database [7]. Additionally, the ability to use custom datasets of sequences allows IgMAT to include unusual antibody sequences. This can be extremely useful to annotate sequences with unusual length or recombination patterns.

**Fig. 1:**
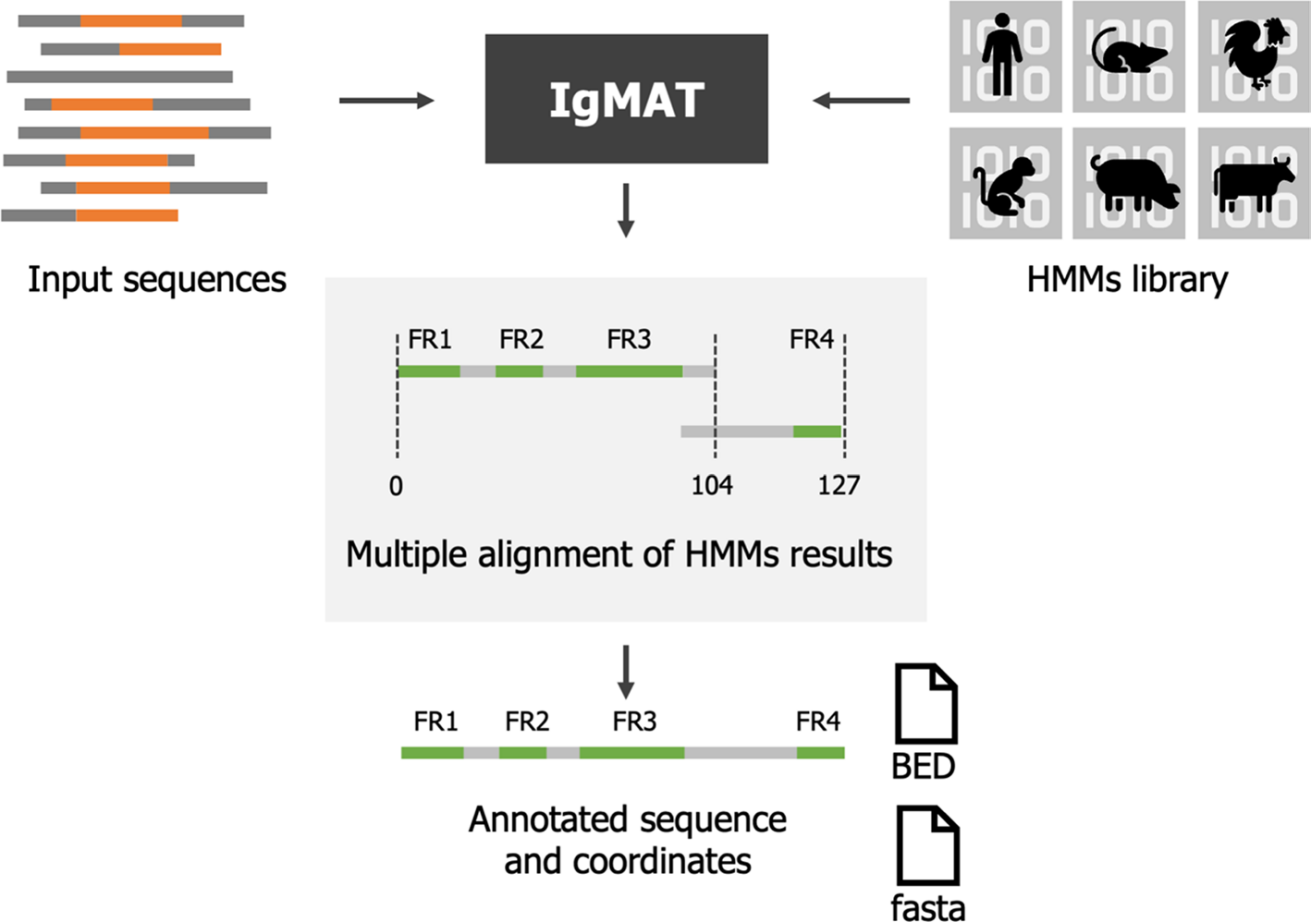
Flowchart illustrating IgMAT pipeline. Input data is supplied in single or multi-fasta files and a set of curated HMM libraries from different species is employed to annotate the input data. Multiple HMM results from the same domain are considered and a consensus sequence is extracted by applying heuristic knowledge of FR and CDR regions, derived from the input model. Results are stored as fasta and BED (Browser Extensible Data) files

IgMAT can annotate single sequences or batches efficiently by distributing the jobs among multiple processes. Each sequence is aligned to the HMMs to find the best matching domain. For the most common antibody sequences, one single match is sufficient to identify all the regions composing the antibody sequence (FR1, CDR1, FR2, CDR2, FR3, CDR3, FR4). However, some antibodies can display ultralong CDR3 sequences or unusual patterns than are not identified by one single match in the HMMs. For this reason, IgMAT considers multiple HMM alignments from the same domain and extracts a consensus sequence that is then validated by applying heuristic knowledge of FR and CDR regions derived from the input model (Fig. 1). This approach allows annotation of most known antibody sequences. However, it is limited by the number and variability of the sequences composing the input dataset, and for some extreme cases it cannot guarantee a proper annotation. To overcome this limitation, IgMAT implements two additional features: a tool for generating custom HMM models and the ability to use a reduced amino acid alphabet.

The default dataset of curated sequences used by IgMAT is generated from IMGT database high-quality germline sequences, that are extracted and aligned by employing a data extraction script based on the code from ANARCI. The generated set of alignments are stored separately in a GitHub repository that is used by IgMAT to build the default model. This organization allows IgMAT to rely on a curated alignment that can be periodically extended and curated. In addition, IgMAT provides the ability to build custom HMM datasets that can be used to annotate sequences. The build tool requires an initial set of FASTA files containing J region and V region sequences separately. From this input set, an alignment is automatically generated from the permutation of all the VJ sequences; after validation of the obtained alignment file, the HMM is created and a name is assigned to the model to be used for the annotation. Optionally, the script can directly accept an alignment file to allow the user to fix any possible annotation error before the HMM model is created.

The ability of IgMAT in recognising and annotating antibody sequences is directly correlated to the coverage provided by the input dataset. If an antibody sequence, or the regions composing it, is not represented by the dataset, it won’t be recognised by the HMM. Reducing the number of amino acids composing the sequence helps simplifying the input data, highlighting chemo-physical patterns that are not properly represented by the input dataset. IgMAT implements the reduced alphabets by Li et al. [10], providing the ability to apply a reduction from 20 amino acids down to three.

## Results

**Fig. 2:**
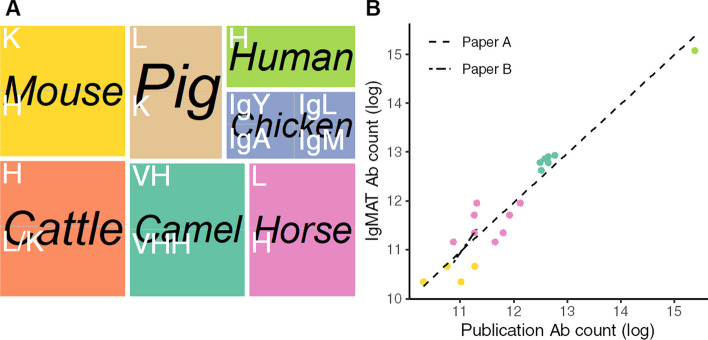
Benchmark dataset composition and evaluation results. **A** The dataset used for testing comprised sequences from different species and isotypes; **B** Comparison of the number of IgMAT annotated antibodies with the antibodies identified in the original papers [6, 11, 12, 14]. Data not available for cattle, chicken, and pig

**Fig. 3:**
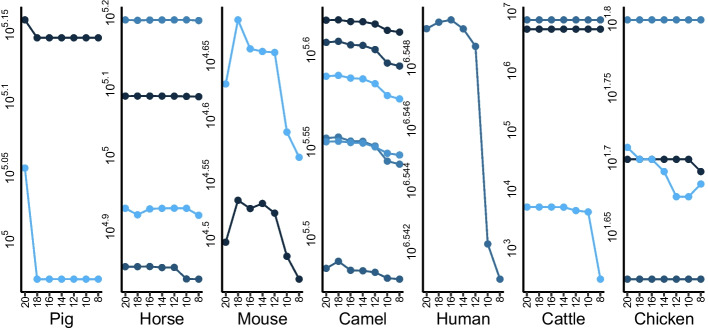
Analysis of Ig datasets with degenerate alphabet. For each analysed dataset, the alphabet was reduced in a range from 20 to 8 and the number of identified Ig sequences was plotted. Data is grouped by species, and different colours are used to distinguish between datasets

IgMAT functionality and versatility was tested by analysing a panel of high-throughput sequencing data, obtained using different technologies, from a range of vertebrate species. Whole repertoire data from horse (*Equus caballus*) [12], mouse (*Mus musculus*) [14], camel (*Camelus bactrianus*) [11], human (*Homo sapiens*) [6], pig (*Sus scrofa*) [15] and chicken (*Gallus gallus*), as well as multiple combined single cell datasets from cattle (*Bos taurus*) [9] were annotated using IgMAT (Fig. 2A, Table 1). The default HMM model (Additional file 1: Table S1) was used for each animal. Input sequences were translated into all 6 reading frames and any sequences containing a stop codon were removed. Over 80% of bovine, camelid, porcine and chicken (except chicken IgA) input sequences were successfully annotated. Horse, mouse, and human annotation rates were similar to those previously found with differences likely due to the different methods used to generate the previously generated data (Fig. 2B). Sequences which failed annotation were checked for valid antibody sequences however the unannotated sequences were either too short (15–20 amino acids) and thus failed alignment or were the incorrect reading frames of valid antibodies which did not contain a stop codon. Overall, IgMAT was able to annotate the overwhelming majority of correct antibody sequences from high-throughput sequencing data from a range of vertebrate species without having to apply tailor-made datasets. The resulting number of sequences annotated for each dataset is in accordance with the original papers [6, 11, 12, 14] (Fig. 2B). The ability to use a degenerate alphabet was tested for each set of data separately, as illustrated in Fig. 3. While the number of annotated Ig decrease with the reduction of the alphabet in pig and cattle, the contrary is true for the other species. In fact, in mouse, camel and human the number of correctly annotated sequences slightly increase when the alphabet is reduced to 18–16 amino acids (Fig. 3). This is probably because the reduced alphabet is able to compensate the HMM dataset poor coverage for some of the species.

**Table 1:** Benchmark of IgMAT annotation

Species	Antibody	Technology	Data Model	Input	Annotated	Previously reported	Ref
Horse1	IgH		EquineIMGT	464260	121106	~ 165000	REF; SRR7653028, SRR7653029
	IgL	Illumina	EquineIMGT	464260	67980	~ 125000	
Horse2	IgH		EquineIMGT	509503	155121	~ 165000	
	IgL		EquineIMGT	509503	83483	~ 125000	
Mouse	IgH	Illumina	Default	36583423	29102	30138	REF; GLDS-141
	IgK		Default	36583423	40585	46904	
Camel1	VH		Default	478669	414554	351209	REF; SRR3544217- SRR3544222
	VHH		Default	467756	403262	309782	
Camel2	VH	Illumina	Default	365029	303845	272373	
	VHH		Default	413897	357825	266320	
Camel3	VH		Default	430736	356061	310279	
	VHH		Default	457564	386652	292751	
Human	IgH	Illumine	Default	8299815	3510922	4796235	REF; SRR11961710-SRR11961728
Porcine	IgL	Roche 454	PorcineIMGT	112214	112212	NA	REF; SRR903523, SRR903581
	IgK		PorcineIMGT	142507	142458	NA	
Chicken	IgM	PacBio	ChickenIMGT	56	49	NA	
	IgA		ChickenIMGT	93	41	NA	
	IgY		ChickenIMGT	67	62	NA	
	IgL		ChickenIMGT	52	48	NA	
Bovine	IgH	Illumina (plus 55 Sanger)	BovineIMGT	5264901	5113752	NA	
	IgL		BovineIMGT	8397687	7445578	NA	

Whole repertoire data from different species were analysed and annotated with IgMAT. Depending on the species, different data models were used. Th default dataset includes human, mouse, rhesus monkey, rabbit, sheep, alpaca, rat and pig IMGT sequences

**Fig. 4:**
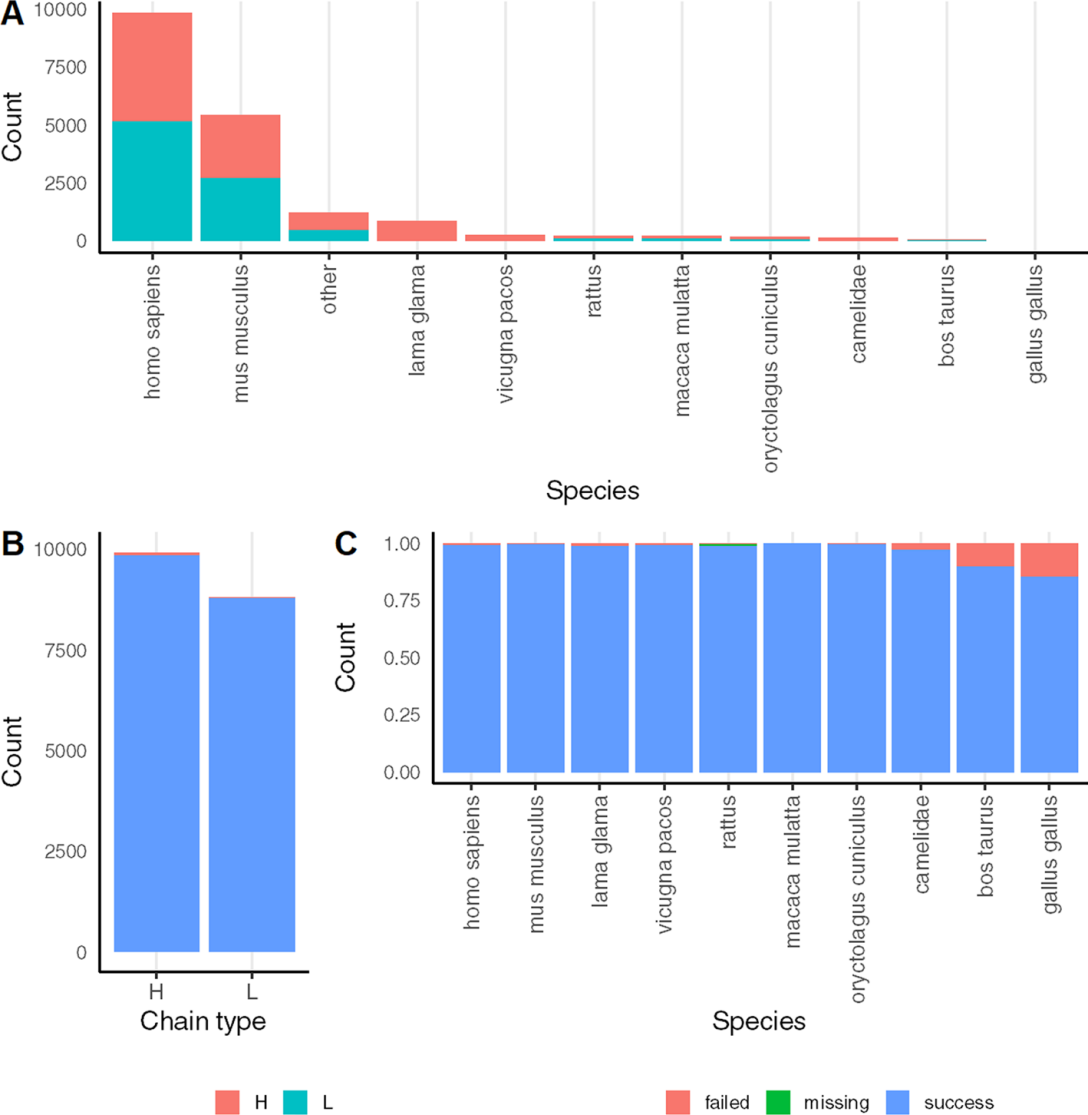
Analysis of PDB dataset. **A** Number of analysed heavy (red) and light (green) chain sequences by species; **B**, **C** Analysis result by chain type and by species. Blue: successfully annotated; Red: failed annotation; Green; missing sequences

In order to test the overall accuracy of the annotation, a dataset composed of sequences extracted from all the experimentally resolved immunoglobulins currently available was analysed. The PDB dataset, containing both heavy and light chains, was extracted from SabDab [5]. This database is a highly reliable source of information that provides antibody annotations derived from the analysis of 3D structures, conveniently numbered according to the IMGT annotation. A total of 18,666 sequences from several different organisms was annotated, including *Homo sapiens*, *Mus musculus*, *Lama glama*, *Macaca mulatta*, *Rattus norvegicus*, *Oryctolagus cuniculus, Gallus gallus* and *Bos taurus,* (Fig. 4A). Sequence annotations were considered passed when the resulting aligned sequence from IgMAT was identical to the one annotated on SabDab, while they were considered missed when no sequence was annotated, and ambiguous when there were mismatches in the resulting IMGT alignment. To ben noted that for most of the ambiguous results, the sequence regions boundaries were correctly annotated. Overall, IgMAT was able to correctly annotate over 100% of the dataset, with 108 sequences correctly regionally annotated with ambiguities in numbering and 5 missed sequences (Fig. 4B, C).

## Conclusion

IgMAT provides enormous flexibility to define custom models incorporating user defined data to better explore antibody repertoires from any vertebrate species, using species specific or multi species databases. Here we have demonstrated an ability to identify and annotate antibody sequences from seven species using only IMGT sequences to build the HMM data set. The addition of sequences from specialist or in house data increases the power to detect the antibody sequences of any species of interest. Additionally, IgMAT streamlines the identification and extraction of antibody variation regions, supporting separate analysis and smooth integration into analysis pipelines. It complements tools like IgBast or MIXCR, which are specialised on analysing T-cell receptor (TCR) and B-cell receptor (CR) nucleotide sequences. The underlying principle of IgMAT allows it to be readily applied to T cell receptor datasets. The program is available under the GPLv3 licence and available to download from GitHub (https://github.com/TPI-Immunogenetics/igmat).

### Availability and requirements

- Project name: IgMAT.
- Project home page: https://github.com/TPI-Immunogenetics/igmat
- Operating system(s): Platform independent.
- Programming language: Python.
- Other requirements: Python 3 or higher.
- License: GPLv3.
- Any restrictions to use by non-academics: none.

### Supplementary Information

**Additional file 1: Table S1**. Dataset composition. Composition of the default HMMs used for repertoire analysis.

## Data Availability

Datasets for the validation of IgMAT were obtained from the original studies and processed into a format suitable for IgMAT analysis. Processed data is available for download from the IgMAT repository.
