# Supplementary material for: IgMAT: immunoglobulin sequence multi-species annotation tool for any species including those with incomplete antibody annotation or unusual characteristics
Source: BMC Bioinformatics. 2023 Dec 21;24:491. doi: 10.1186/s12859-023-05624-2 (PMC10740263; doi:10.1186/s12859-023-05624-2)
Supplement: Supplementary file 1 — Additional file 1: Table S1. Dataset composition. Composition of the default HMMs used for repertoire analysis. [file 12859_2023_5624_MOESM1_ESM.docx]

TABLES

| **Scientific name** | **Alphabet size** | **Heavy** | **Kappa** | **Lambda** |
| --- | --- | --- | --- | --- |
| Homo sapiens | 20 | 294 | 195 | 150 |
| Mus musculus | 20 | 584 | 404 | 12 |
| Rattus norvegicus | 20 | - | 276 | 12 |
| Oryctolagus cuniculus | 20 | 228 | 16 | 38 |
| Macaca mulatta | 20 | 574 | 74 | 483 |
| Sus scrofa | 20 | 65 | 35 | 12 |
| Vicugna pacos | 20 | 30 | - | - |
| Bos taurus | 20 | 36 | 6 | 95 |
| Ovis aries | 20 | - | 5 | 48 |

**Table S1 Dataset composition**. Composition of the default HMMs used for repertoire analysis.
